# Clinical study to assess influence of immediate provisionalization and various implant morphologies on implant stability: A prospective clinical study

**DOI:** 10.3389/fsurg.2022.1095741

**Published:** 2023-01-06

**Authors:** Meiyao Qi, Shiyong Deng, Zhen Tan

**Affiliations:** ^1^ State Key Laboratory of Oral Diseases, National Clinical Research Center of Oral Diseases, West China Hospital of Stomatology, Sichuan University, Chengdu, China; ^2^ Department of Implantology, West China Hospital of Stomatology, Sichuan University, Chengdu, China

**Keywords:** implant therapy, implant stability quotient, resonance frequency analysis, immediate provisionalization, clinical study

## Abstract

**Introduction:**

The aim of this study was to evaluate the influence of different implant morphologies and immediate provisionalization options on the change of implant stability.

**Methods:**

94 Patients were randomized to receive implants from Straumann^®^ BL/Straumann^®^ BLT/Astra OsseoSpeed^®^ TX, meanwhile having the same opportunity to receive healing abutment or immediate provisionalization. Implant stability quotient (ISQ) and marginal bone loss (MBL) were recorded at following timepoints. Parametric statistic was used for data analysis.

**Results:**

Data showed that ISQ and MBL values of conical/straight/straight with micro-thread neck implants had no significant difference.

**Discussion:**

Immediate provisionalization options could move the dip point of ISQ values ahead or delayed around one week, which were also relevant to implant systems. MBL values were proved to be unaffected by both two factors mentioned above.

## Introduction

Recently, implant-supported maxillofacial prostheses have become one of the most favored methods to cope with congenital craniofacial deformities, chosen by both patients and surgeons. This therapeutic regimen is frequently applied in multiple areas of oral and maxillofacial surgery, including reconstruction of the maxillofacial skeleton, restoration of hemifacial/small mandibular deformity, temporomandibular joint (TMJ) total joint replacement, and orthognathic surgery (1, 2). As a kind of restoration and/or replacement of stomatognathic and associated facial structures by artificial substitutes, implant-supported prostheses not only help disfigured and socially unacceptable people to regain a normal life with normal craniofacial appearance but also help reconstructing their facial esthetics, oral function, social-acceptance, and self-confidence (3). The stability of implants is the guarantee of a long-term successful treatment.

Achieving good primary and secondary stability after implantation is one of the necessary conditions for realizing the functional load of the implant, and it is the decisive factor for whether it can bear the functional load and affect the long-term stability. Therefore, the evaluation of implant stability after implantation is of great significance.

Implant stability testing methods are as follows, including bone-implant-contact (BIC) histomorphological analysis, mechanical test, impact test, implant insertion torque (IT) test, periodic inspection value test, x-ray follow-up, and resonance frequency analysis (RFA) (4, 5). Among them, BIC histomorphological analysis (bone to implant contact analysis) is considered to be the gold standard, but its application conditions are limited. The most commonly used ones clinically are IT and RFA. IT is a measure of the frictional resistance that the implant encounters through the axial rotational movement of the top. RFA records the peak amplitude of the vibration response to small sinusoidal signals through a magnetic device fixed on the implant and encodes it as an implant stability quotient (ISQ). ISQ is an objective index reflecting the stiffness of the bone-implant system. As the stiffness of the bone-implant interface increases, so does the ISQ (5). A series of studies have proved the repeatability and accuracy of the Osstell system in measuring ISQ values (6–8).

ISQ varies with the progression of implant-bone osseointegration. During osseointegration, the initial mechanical stability is gradually replaced by biological stability. It is generally believed that with the decrease of initial stability and the increase of secondary stability, ISQ will experience a process of decrease and then increase. The lowest value of ISQ appears at 4–5 weeks after operation, in general, while it is not fixed among different implant systems and may appear in the third or seventh week (9, 10). The literature suggests that implants with failed osseointegration will have a significantly lower ISQ (4). ISQ < 36 was significantly associated with implant failure, but not with overall implant survival (11, 12). Due to its low predictive sensitivity, ISQ cannot be used as a reference indicator for implant failure (13).

The jaw position of the implant has a significant impact on ISQ. Systematic review shows that the ISQ of mandibular implants was significantly higher than that of maxillary implants, and the difference was statistically significant (4). There are also significant differences in ISQ between different restoration methods. For example, the ISQ of fixed denture restoration is significantly higher than that of overdenture restoration (4) and progressive provisionalization (immediate provisionalization without abutment and occlusal contact, adjacent contact at 1 month after surgery, and occlusal contact at 2 months after surgery). Median occlusal contact increased faster than the delayed load ISQ (14).

The effect of different implant morphologies on ISQ has also been studied. Different implant systems often have different thread spacing, implant morphology, microscopic topography, and surface modification methods, which may affect the mechanical retention force and osseointegration efficiency of the implant. A randomized controlled trial demonstrated that different implant neck designs affected ISQ changes within 6 months after implantation (15). The ISQ value and change in trend of implants of different systems are slightly different (16). Implants with wider thread spacing may have higher initial ISQ than implants with narrow thread spacing, and this difference disappears after 90 days (17). There are also differences in the ISQ curve between bone-level implants and soft-tissue-level implants (9).

The aim of this study was to investigate the difference in the trend of implant stability measured as ISQ among ASTRA TX, Straumann BL, and Straumann BLT systems with various implant morphologies. Another aim of this study was to determine whether a relation exists between the ISQ and provisional options. Further investigation considered the effect of implant morphologies and surface modifications on marginal bone loss (MBL).

## Materials and methods

### Patient selection

Patients with missing teeth in upper or lower jaw who were allocated for implant placement were recruited at the Department of Implantology, West China School of Stomatology, Sichuan University, between September 2020 and December 2021.

Inclusion criteria were as follows: (a) aged between 18 and 70 years; (b) generally healthy, without systemic diseases; (c) 3 months or more after teeth extraction, requiring implant-support fix restoration,; (d) sound oral hygiene and stable periodontal status; (e) without surgical contraindications or anesthesia contraindications; (f) possibility for a one-stage implant with transmucosal healing, without any need of bone augmentation; (g) the ability and willingness to comply with all study requirements and agree to observation till final restoration; (h) self-signed informed consent.

Exclusion criteria were as follows: (a) moderate or heavy smokers (≥10 cigarettes per day); (b) patients suffering from severe bruxism; (c) history of head and neck radiotherapy, chemotherapy, or bisphosphonate treatment; (d) pregnant or lactating females; (e) poor oral hygiene and unstable periodontal condition; (f) alcoholism; (g) uncontrolled diabetes, osteoporosis, rheumatic arthritis, oral cancer, or neoplasm; (h) a need for bone grafting in the implant site; (i) insertion torque under 20 N cm (due to the need to remove the healing abutment/provisional teeth several time during the healing period).

The study protocol and the consent forms were approved by the Ethics Committee of West China School of Stomatology (WCHSIRB-CT-2022-250), Sichuan University, and the guidelines for Good Clinical Practice were respected. All participating individuals were informed of the risks and benefits as well as the procedures of the study; they all gave written informed consent. The study was investigator-initiated and was supported by an unconditional grant from Sichuan University. The study was in compliance with the EQUATOR guidelines for clinical studies.

### Study design

#### Implants and surgical procedure

All recruited patients should go through preoperation examination, including the following: (1) Basic health data acquisition, preoperative blood test, and blood pressure measurement to exclude contraindications; (2) cone-beam computed tomography (CBCT) examination to obtain hard tissue data; (3) intraoral photographs to obtain soft tissue condition. Then, the principal investigator of the project would make a treatment plan for each patient before surgery, ensuring the patient's oral condition was suitable for implant placement surgery and restoration without necessity of bone augmentation, after which the consent form would be signed by the patient himself/herself.

**Figure 1:**
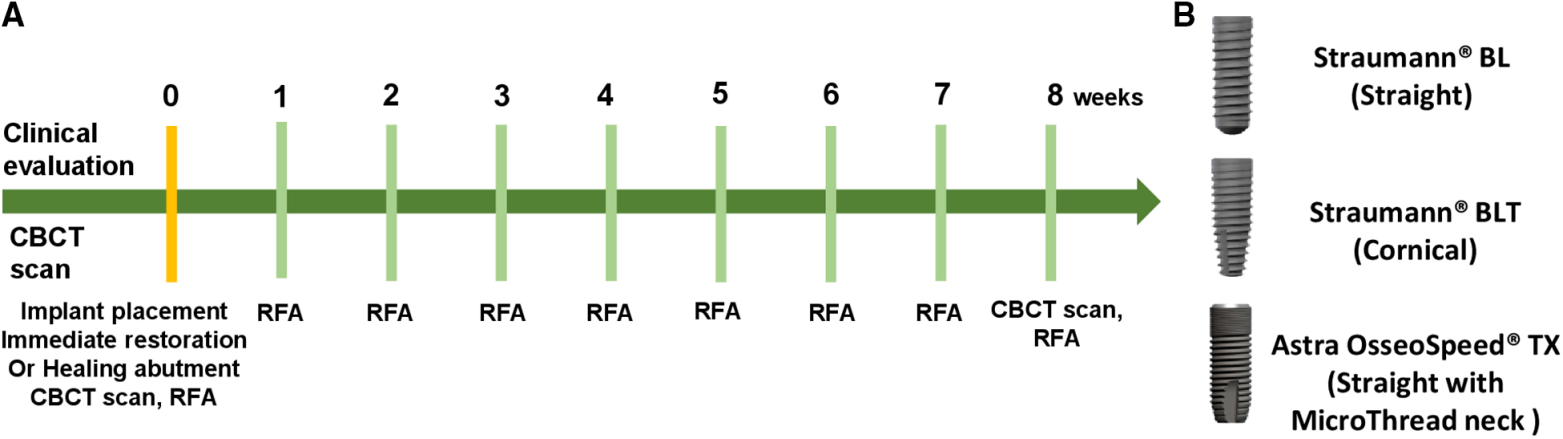
(**A**) Implant stability parameters and data collection time points. (**B**) Implants with different morphologies used in the study: Straumann® BLT/Straumann® BL/ASTRA OsseoSpeed® TX. All implants are with a tapered design.

All patients received a dental implant in the missing site be means of a one-stage transmucosal approach with healing abutment or immediate provisional restoration. Prior to surgery, patients were randomly allocated to Straumann Bone Level, Straumann Bone Level Tapered, and ASTRA OsseoSpeed TX groups, while in each group provisionalization timing were stochastically chosen (immediate provisionalization or nonimmediate provisionalization). All the surgical procedures were performed by one certified surgeon (ZT) using the same surgical technique and protocols. Three different commercially available tapered dental implants were used (Figure 1): Straumann Bone Level, Straumann Bone Level Tapered, and ASTRA OsseoSpeed TX. Healing abutments or temporary abutments of the same system as the implants were applied after surgery. Implants from every individual system are manufactured using the same titanium alloy and have identical surface characteristics.

Antibiotics were used prophylactically for 0.5–1 h before surgery. Every patient was asked to do mouth rinsing with 0.2% chlorhexidine solution for 3 min. Surgery was performed under local infiltration anesthesia with articaine and epinephrine. Following local anesthesia, the implant site was exposed *via* a mid-crestal incision and a full-thickness flap was elevated. Positions of the implants were marked with a round bur. The surgical procedure followed the recommendation of the manufacturers. After preparation of the osteotomy site, the clinical records of implant diameter, length and implant torque, and ISQ values were recorded, and a healing abutment or an immediate restoration was randomly placed.

(1) Immediate provisionalization group: The three positions of the implant were obtained by intraoral pick-up to make a temporary restoration. Immediately after the operation, the immediate restoration was inserted and adjusted to make it in the median jaw position, forward extension and lateral movement. There was no contact with the opposite teeth and adjacent teeth, and the wounds were closed tightly with sutures.
(2) Nonimmediate provisionalization group: The wound was closed tightly after the healing abutment was installed.

Immediate treatment after operation included the following: (1) taking CBCT to verify the three-dimensional position of implant placement; (2) taking 2 g amoxicillin (600 mg clindamycin for allergic patients) every 8 h, and 600 mg diclofenac sodium twice a day to relieve pain for 7 consecutive days after operation; (3) rinsing with 0.2% chlorhexidine solution daily for 14 days after surgery to prevent infection.

#### Clinical measurements and follow-up

The maximal insertion torque value of each implant was approximately reported and recorded by the surgery performer. All RFA measurements were carried out using the Osstell resonance frequency analyzer (Integration Diagnostics, Sweden), by connecting directly to the implant. A total of nine ISQ measurements were taken per implant per time point: three from buccal, three from mesial, and three from the proximal side.

From the surgery (0 week), resonance frequency analysis measurements were obtained in the same way at 7, 14, 21, 28, 35, 42, 49, and 56 days (1, 2, 3, 4, 5 ,6, 7, and 8 weeks, respectively) following implant placement; the implant stability quotient was measured at the buccal, lingual, and proximal sides. At each follow-up evaluation, the healing abutment was removed and cleaned. The SmartPeg was inserted into the implant, and the ISQ measurements were taken. The peg was then removed, and the healing abutment or provisional restoration was replaced.

At the final examination day (day 56), a second digitized CBCT examination was taken, prior to implant restoration. MBL around each implant was assessed using the radiograph acquisition program by measuring the distances between the implant platform and the most coronal bone contact with the implant; implant diameter was used for internal calibration. This allowed a millimetric assessment of the actual bone loss. The differences between the baseline CBCT (postoperative) and the final CBCT were used for data analysis.

### Statistical analysis

The statistical analyses performed included Student's *t*-test for independent samples and a one-way ANOVA and Tukey's procedure for multiple pairwise comparisons. Mann–Whitney, Wilcoxon, and Friedman tests were used when nonparametric alternatives were required. Statistical correlations were analyzed with Pearson's coefficient and Spearman's rank correlation test.

Multivariate linear regression was used to determine how much of the variation in each outcome variable was accounted for by each predictor variable. The categorical predictor variable (bone type; more than two categories) was converted into two binary dummy variables, one for each original predictor variable category. This procedure was unnecessary for regression analysis with the two binary independent variables diameter and length. All statistical analyses were performed by an experienced dental statistician using IBM SPSS Statistics v. 22.0 software.

## Results

**Figure 2:**
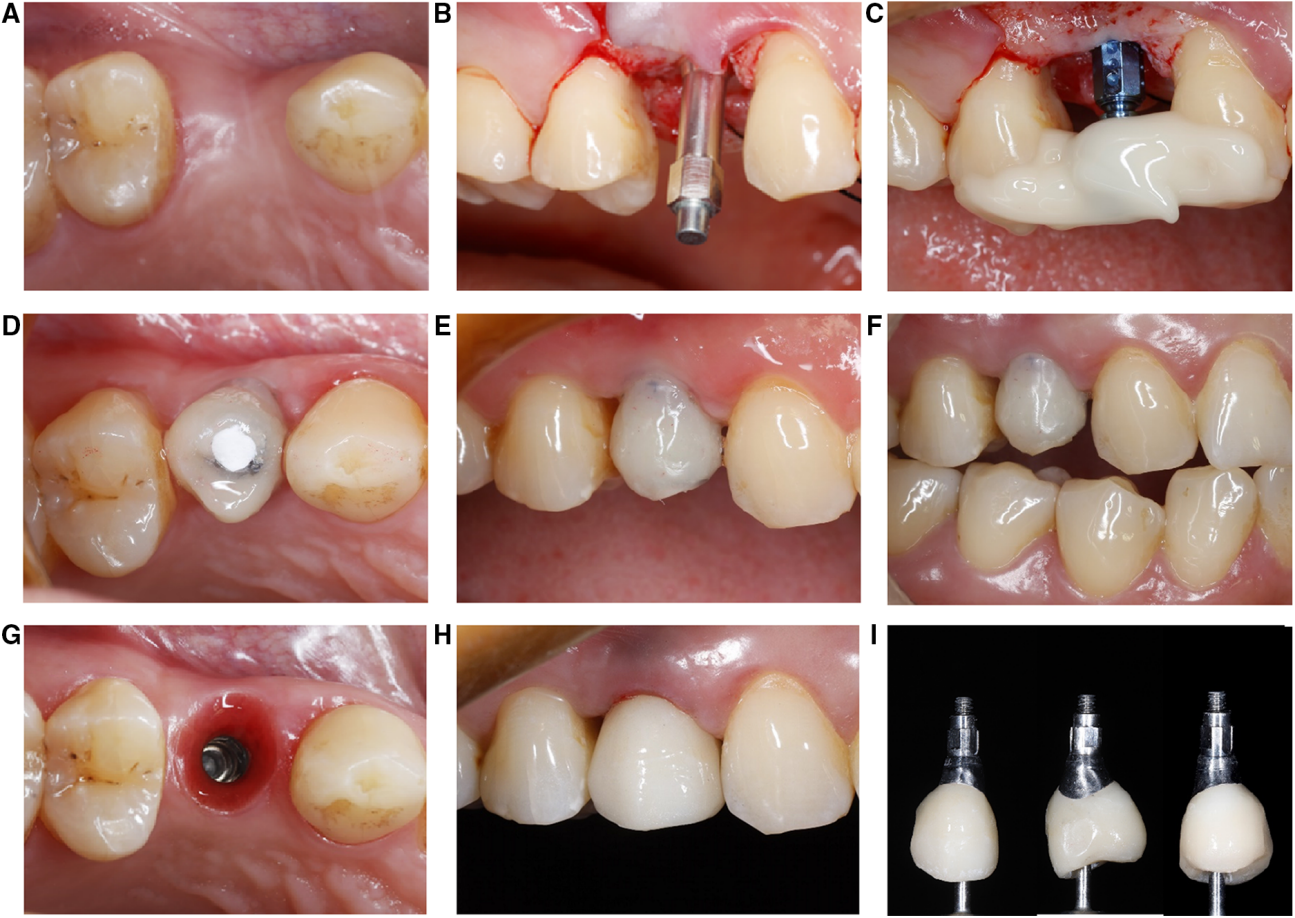
Clinical images of experimental sequences (immediate provisionalization group). (**A**) Implant recipient site. (**B**) An RFA transducer attached to the implant body to measure ISQ value. (**C**) On-site pick-up to make chair side immediate provisionalization. (**D–F**) Immediate provisionalization restored. (**G–I**) Final restoration marked the end of the study period. RFA, resonance frequency analysis; ISQ, implant stability quotient.

Patients who arrived at the hospital dental clinic seeking restoration of missing tooth and have the right dimensions of the implant site in their CT imaging were offered to participate in the study. A total of 94 patients (46 females, 48 males) received dental implant-supported restoration. Seventeen patients received Straumann® BL implants (Straumann® BL group), 15 patients received Straumann® BL implants with immediate temporary restoration (Straumann® BL immediate provisionalization group), 15 patients received Straumann® BLT implants (Straumann® BLT group), 15 patients received Straumann® BLT implants with immediate temporary restoration (Straumann® BLT immediate provisionalization group), 15 patients received ASTRA OsseoSpeed® TX (OsseoSpeed® TX group), and 17 patients received ASTRA OsseoSpeed® TX implants with immediate temporary restoration (ASTRA® TX immediate provisionalization group). Most implant placement procedures were completed, except for one adverse event [one patient in the ASTRA TX immediate provisional group presented low insertion torque value (lower than 20 N cm) and was excluded from the longitudinal RFA analysis, due to the exclusion criteria]. Implant stability parameters and data collection time points are shown in Figure 1A, with the three different implant morphologies pictured in Figure 1B. All implants achieved a sufficient insertion torque. Figure 2 exhibited the whole treatment sequences of one patient in the immediate provisionalization group, including receiving surgery, doing RFA measurements, receiving immediate provisionalization, and final restoration.

The demographic characteristics of the patients were comparable between groups, and they are presented in Table 1. Of the 94 implants studied, 47 (50%) were inserted in women and the remaining 47 (50%) were in men. Patients’ age ranged between 19 and 68 years with a mean of 49 years.

**Table 1:** Patient and implant demographic characteristics.

	Straumann® BL	Straumann® BL immediate provisionalization	Straumann® BLT	Straumann® BLT immediate provisionalization	ASTRA® TX	ASTRA OsseoSpeed® TX immediate provisionalization
Patient gender (M/F)	9/8	6/9	8/7	7/8	7/8	10/7
Patient age (years)	47 ± 23 (range: 19–63)	38 ± 31 (range: 22–63)	46 ± 22 (range: 21–68)	46 ± 24 (range: 22–70)	45 ± 21 (range: 24–67)	39 ± 29 (range: 20–68)

With respect to implants position and numbers (Table 2), 34 (36%) implants were put in the maxilla, while the rest 60 (64%) were inserted in the mandible. Implants put in the site of central/lateral incisors, canines, premolars, and molars were 5 (5%), 5 (5%), 22 (23%), and 62 (66%), respectively.

**Table 2:** Implants position and numbers.

	Implant position and numbers (BL + BLT + TX = 94)		
	Straumann® BL	Straumann® BLT	ASTRA OsseoSpeed® TX
Upper/lower	14/18	10/20	10/22
Central incisor	0	2	0
Lateral incisor	1	0	2
Canine	0	4	1
Premolar	11	4	7
Molar	20	20	22

### RFA measurements—ISQ analysis

On surgery (day 0), according to Table 3, the mean ISQ of three implant systems (immediate provisionalization or nonimmediate provisionalization) were 78.24/81.65 (Straumann® BL, SD = 4.83/4.23), 77.86/81.29 (Straumann® BLT, SD = 5.20/6.05), and 79.30/82.48 (ASTRA OsseoSpeed® TX, SD = 5.27/4.87), exhibiting no significant difference. In general, ISQ of day 56 (secondary ISQ) postop was statistically significantly higher than the measurements recorded for days 0 (primary ISQ). Little variation of ISQ values, among the three different-morphology implant groups, were observed, indicating that ISQ values mainly change accordingly to healing status (primary and secondary). Therefore, conclusions can be reached that under the same surgical procedure, implant morphologies did not seem to affect ISQ value much.

**Table 3:** Mean and SDs of three implant systems in terms of maximum insertion torque and RFA (*p* < 0.05, one-way ANOVA).

	Implant System	*n*	Mean	SD	Significance (*p*)
RFA (ISQ) (immediately postop)	Straumann® BL	32	78.24	4.83	0.511
	Straumann® BLT	30	77.86	5.20	
	ASTRA OsseoSpeed® TX	32	79.30	5.27	
RFA (ISQ) (56 days postop)	Straumann® BL	32	81.65	4.23	0.638
	Straumann® BLT	30	81.29	6.05	
	ASTRA OsseoSpeed® TX	32	82.48	4.87	

SD, standard deviation; RFA, resonance frequency analysis; ISQ, implant stability quotient.

**Figure 3:**
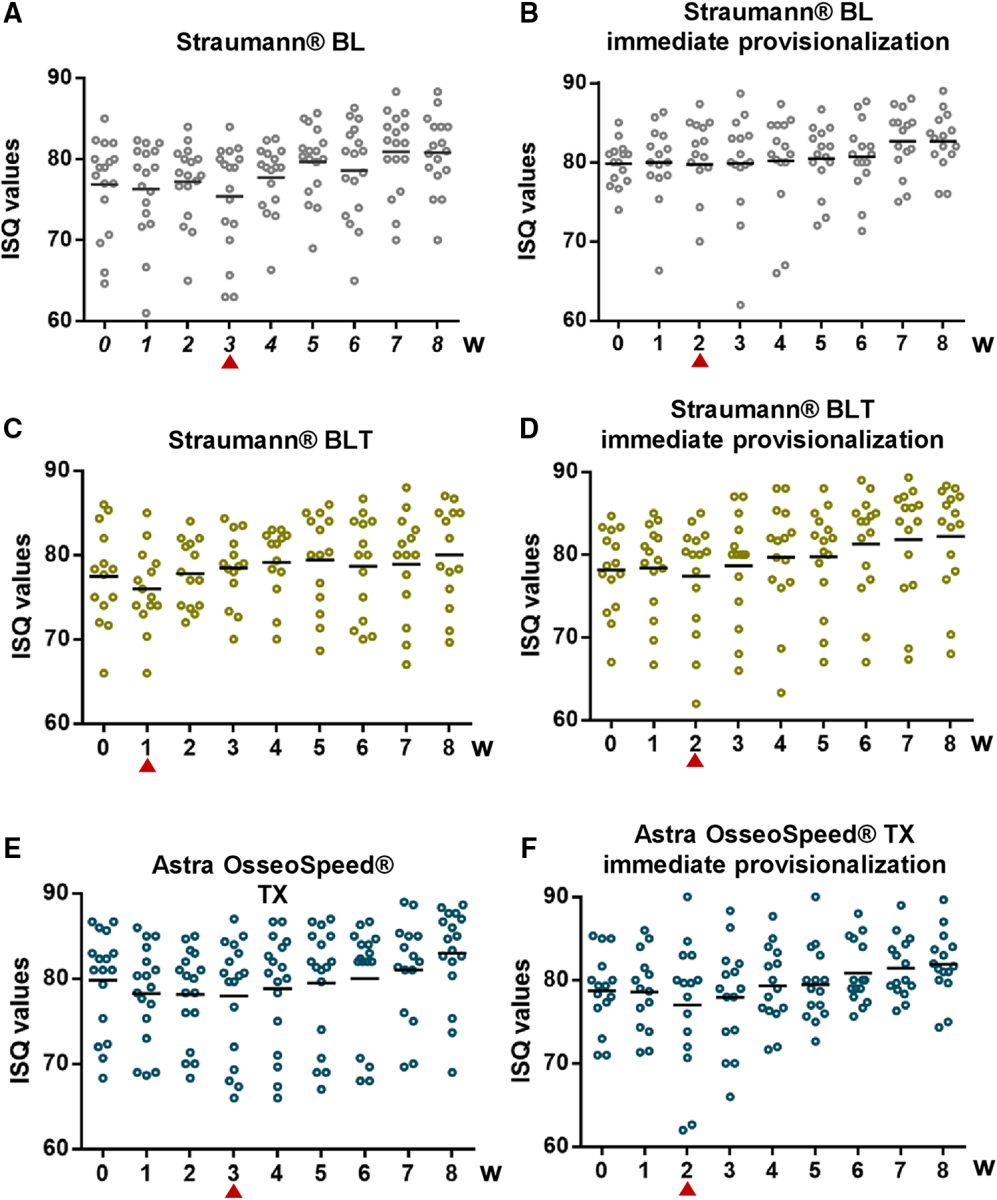
Mean ISQ values of each time point during examination period, lowest implant stability quotient (ISQ) points marked by red triangles. (**A-F**) All six groups of different implant morphologies and immediate provisionalization options.

In terms of immediate provisionalization choice, ISQ value at day 14 exhibited significant difference between the immediate provisionalization group and the nonimmediate provisionalization group (mean ISQ values 76.69/79.27, SD = 5.32/4.78), which reminded us that at the early stage (14 postop), a more careful attitude should be taken facing implants with immediate provisionalization. Unnecessary operation should be avoided at the first 2–3 weeks after implantation (Table 4). During the whole healing period, the RFA values fluctuated and showed an overall trend of declining first and then rising later. It was worth noting that, at the time point of 7, 14, and 28 days (1, 2, and 3 weeks after implantation, respectively), ISQ values fell to the bottom of the whole period, though without statistical significance (Figure 3). An interesting finding was that all three implant groups showed a similar pattern of stability change over time; there was a drop in ISQ among the first 1–3 weeks, and then, the ISQ gradually increased back until reaching the top at the end of the study (day 56, week 8). /the Straumann® BLT group exhibited the earliest bottom ISQ time point at week 1, while the other five groups varied little, indicating us that during 1–3 weeks, implants should be handled with extra caution.

**Table 4:** Mean and SD of immediate provisionalization group and nonimmediate provisionalization group in terms of maximum insertion torque and RFA (*p* < 0.05, Student's *t*-test for independent samples).

	Immediate provisionalization	*n*	Mean	SD	Significance (*p*)
RFA (ISQ) (14 days postop)	Yes	47	76.69	5.31	0.047
	No	47	79.27	4.78	
RFA (ISQ) (56 days postop)	Yes	47	81.01	4.80	0.124
	No	47	82.62	5.22	

SD, standard deviation; RFA, resonance frequency analysis; ISQ, implant stability quotient.

**Figure 4:**
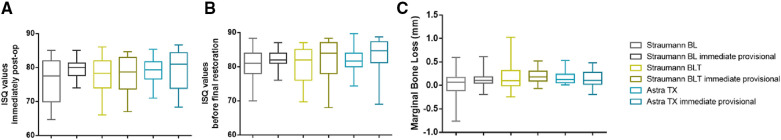
(**A,B**) ISQ measurements at immediately post-surgery (day 0) and before final restoration (day 56) in the six groups. Boxes denote quartile range of measurements. (**C**) Mean MBL values of the six groups (whiskers—95% confidence intervals). MBL, marginal bone loss.

In order to cast light on the variation of ISQ values at the first and last time points, the Pearson correlation coefficient (PCC) was analyzed. However, results showed that the two factors that we were interested in (different implant morphologies and immediate provisionalization choice) exhibited minor influence on the ISQ values at days 0 and 56 (Tables 5, 6). Intuitive differences between groups are shown in Figures 4A,B.

**Table 5:** Mean of the RFA immediately after insertion and after 3 months and the PCC (*r*).

Implant system	*n*	Mean RFA (ISQ) (immediately postop)	Mean RFA (ISQ) (3 months postop)	PCC (*r*)
Total	94	78.48	81.82	0.524
Straumann® BL	32	78.24	81.65	0.639
Straumann® BLT	30	77.86	81.29	0.520
ASTRA OsseoSpeed® TX	32	79.30	82.48	0.432

PCC, Pearson correlation coefficient; RFA, resonance frequency analysis; ISQ, implant stability quotient.

**Table 6:** Mean and the PCC (*r*) of the RFA of immediate provisionalization group and nonimmediate provisionalization group.

Immediate provisionalization	*N*	Mean RFA (ISQ) (immediately postop)	Mean RFA (ISQ) (3 months postop)	PCC (*r*)
Total	94	78.48	81.82	0.524
Yes	47	77.69	81.01	0.421
No	47	79.27	82.62	0.610

PCC, Pearson correlation coefficient; RFA, resonance frequency analysis; ISQ, implant stability quotient.

### Radiographic measurements—MBL analysis

The average radiographic MBL was measured at the final CBCT taken on day 56 compared with day 0; the mean bone loss for the Straumann® BL/Straumann® BLT/ASTRA OsseoSpeed® TX group was 0.08/0.19/0.16 mm (SD = 0.26/0.24/0.16). There was no statistical significance (*p* = 0.1114) among the different implant design groups, from which we could speculate that implant morphologies did not affect MBL much (Table 7). As for immediate provisional and nonimmediate provisional groups, average MBL was 0.16/0.12 mm (SD = 0.18/0.27). MBL amount was slightly higher in the immediate provisionalization group (Table 8), still without statistical significance (*p* = 0.459). Intuitive differences between groups are shown in Figure 4C.

**Table 7:** Mean and SD of three implant systems in terms of MBL (*p* < 0.05, one-way ANOVA).

	Implant system	*N*	Mean	SD	Significance (*p*)
MBL	Straumann® BL	32	0.08	0.26	0.114
	Straumann® BLT	30	0.19	0.24	
	ASTRA OsseoSpeed® TX	32	0.16	0.16	

SD, standard deviation; MBL, marginal bone loss.

**Table 8:** Mean and SD of immediate provisionalization group and nonimmediate provisionalization group in terms of MBL (*p* < 0.05, Student's *t-*test for independent samples).

	Immediate provisionalization	*N*	Mean	SD	Significance (*p*)
MBL	Yes	47	0.16	0.18	0.459
	No	47	0.12	0.27	

SD, standard deviation; MBL, marginal bone loss.

## Discussion

Nowadays, congenital craniofacial deformities, causing bone defects, misalignment, and soft tissue asymmetry, often lead to severe defects of related soft and hard tissues, which cause serious obstacles to patients’ speech, swallowing, chewing, breathing, and other functions, and severely affect patients’ physiology and psychology. Commonly used methods for craniofacial defect repair include prosthesis repair, bone graft repair, and individualized implant repair, among which the dental-supported prosthesis repair has been widely used, while its postoperative functional recovery is proved to be more than acceptable. Implant-supported prostheses with different structural designs have been gradually applied in clinical practice.

Implant stability is considered one of the most essential factors that affect the long-term survival rate. In this study, ISQ values and trend of change were similar to previous clinical reports (18, 19). Thickness of the cortical bone, cortical-and-cancellous ratios, implant morphologies, and immediate/late provisionalization options all proved to play a role in regulating implant stability (20). The changing trend of ISQ values were mainly reported to have a bottom period around week 2–4 after implant insertion, which were quite similar to the results in this study (week 1–3) (4).

As for different implant morphologies, previous studies proved that both straight and conical implants had approximately the same lowest ISQ values period, which was around 1–3 weeks after surgery. In our study, the same results were reached by parameter analysis. Rotation force should not be applied onto the implants, or unexpected situation might happen. With respect to three implant morphologies (straight/conical/straight with a microthread neck), the bottom point of ISQ value curve came 1 week later (2–3 weeks) in the group of straight and straight with microthread neck implants, while the conical implant groups had their dip point 1 week earlier (1–2 weeks). This characteristic difference can be owned mainly to the conical shape and narrowed root diameter and, probably, the unique surface modification (21–23).

In recent decades, on behalf of higher esthetic requirements and improvements in chair-side restoration techniques, immediate provisionalization, from single-tooth implant restorations and full-arch restorations, have gained intense attention and popularity (24). Studies have been made to investigate different patterns of osseointegration in the immediate and nonimmediate provisionalization group, which mainly laid in the pattern of dynamic or static interfacial activities (25). Implant stability has long been identified as a prerequisite for osseointegration (26). Therefore, losing stability would be disastrous for restoration effect, leading to early failure and unsatisfied doctor–patient relationship. As one of the most important prerequisites in immediate provisionalization, achieving high primary implant stability among groups should not be neglected.

As for the influence of the immediate provisionalization on the ISQ values, it still remained unclear (27, 28). From our findings in this study, in the straight implant groups (Straumann BL and ASTRA TX, with or without immediate provisionalization), ISQ value dip points intended to occur 1 week earlier among the immediate provisionalization group, indicating that the most hazard period of implant survival had been brought 1 week forward by the immediate provisionalization process. However, things turned out slightly different among the conical implant groups (Straumann BLT, with or without immediate provisionalization). The dip points of ISQ values tended to delay approximately 1 week in the immediate provisionalization group. So far, there have been no literature studies focusing on the influence of immediate provisionalization on the change of ISQ values. We boldly speculated from our previous results that the immediate provisionalization process played a crucial role in affecting the change, while the dip points of ISQ moving beforehand or afterward were majorly decided by the morphology of implants. The stability of straight or columnized implants with immediate provisionalization was achieved 1 week later. Nevertheless, it seemed that the stability of conical implants with immediate provisionalization was obtained 1 week earlier than the group without immediate provisionalization. To conclude, it could enlighten implant practitioners that when using conical implants, designing immediate provisionalization should be a way to obtain acceptable ISQ values earlier than usual, even though the mechanism behind this remained undiscovered.

Marginal bone loss has been proved to be associated with loads of factors (25, 29). According to Simmons et al., surgical techniques, insertion torques, provisionalization options, and implant designs all contribute to the variation (19). In this study, factors such as surgical procedures and insertion torques had already been constricted; implant morphologies and provisionalization options were the major influencers (30). The amount of marginal bone loss for implants with three different morphologies all fell within the acceptable range. We observed the highest MBL in conical implants (0.19 mm) after 9 weeks, and then came the straight implants (0.16 and 0.08 mm). However, there were no statistically significant differences between these groups. This indistinctive difference in MBL could possibly be owned to the unique structure of conical implants, whose necks were supposed to yield more compressive force than the straight ones (31, 32), and then accelerating bone loss around implant marginal areas. This conclusion also clicked with findings published by Atieh et al. that parallel-walled dental implants appeared to slow down marginal bone loss (33).

This study proved that the difference of ISQ values and MBL among implant groups with differed morphologies is minor. Ensuring the premise of implantation torque, straight implants (with or without microthread neck design), and conical implants were all suitable for immediate provisionalization with a relatively high success rate. However, during the dip periods of ISQ values, which varied among groups, more care should be taken into our treatment procedure. Limitation of this study was that longer term of observation should be performed. Further studies are required to make certain the mechanism behind the different ISQ trend of dental implants with varied morphologies.

## Data Availability

The raw data supporting the conclusions of this article will be made available by the authors, without undue reservation.
